# Phytostabilizing challenging mine wastes at the cost of phytostabilization

**DOI:** 10.1186/s12302-017-0110-4

**Published:** 2017-03-06

**Authors:** Xiaofang Li

**Affiliations:** Key Laboratory of Agricultural Water Resources, Centre for Agricultural Resources Research, Institute of Genetics and Developmental Biology, Chinese Academy of Sciences, 050021 Shijiazhuang, China

From time to time, tailings dam failures occur in most mining nations, such as the disaster in Minas Gerais, Brazil in 2015 (Science, 18 Dec 2015) and more recently the one in Luoyang in China (http://zy.takungpao.com/2016/0810/270511.html). This has raised a concern on the safe storage of mine tailings in tropical mining nations, where extreme weathers are inevitable, as well as in other parts of the world. Strengthened policing is obviously urgently needed in these areas to guide the design and monitoring of tailings storage facilities, and to balance the environmental protection and mining profit. Yet, we need to admit that it is still challenging worldwide to stabilize surface tailings, particularly those of base metal mines, at a reasonable cost, even if a safe geotechnical structure is achieved.

One of such challenges facing restorationists is how to stabilize tailings using plants cost-efficiently (i.e. phytostabilization [1]). It is commonsense that a vegetation cover is the best option to minimize soil surface erosion by wind and rainfall. The advent of phytostabilization technologies is motivated by the fact that the volumes of legacy mine tailings have increased drastically in the past decades and the use of huge amount of capping soils by conventional dry cover technology has been impractical.

In some developing countries like China, cost-effective phytostabilization technologies are more in need. Till 2012, China has accumulated more than 12,000 tailings impoundments, 3000 of which are with a volume greater than 10^6^ m^3^. Unfortunately, most of the inactive impoundments, particularly the smaller ones, have not been properly closed. Many have even unknown liability subjects [2]. Worse, China’s tailings impoundments are mostly located in densely populated areas, e.g. the central China and northeast China, and many are just adjacent to arable land. This complicates the management, and the proper closure of these tailings ponds has been an economic burden to the nation.

I thus propose that the development of cost-effective phytostabilization technologies (e.g. the technosol technologies [3]) can be a priority for the proper management of legacy mine tailings in China. The progress in technosol technologies may lead to the success of converting the troublesome mine tailings to valuable land resources, that is, phytostabilizing the mine wastes at the cost of phytostabilization in the near future.
